# Sacubitril/valsartan mitigates cisplatin-induced liver injury through modulation of oxidative stress, Caspase-3 activity, and RXR-α signaling in experimental rats

**DOI:** 10.3389/fphar.2026.1745023

**Published:** 2026-02-05

**Authors:** Majed N. Alshammari, Ahmad H. Alhowail, Mohamed S. Abdel-Bakky, Maha A. Aldubayan

**Affiliations:** Department of Pharmacology and Toxicology, College of Pharmacy, Qassim University, Buraydah, Saudi Arabia

**Keywords:** retinoid X receptor-alpha, caspase-3– mediated apoptosis, cisplatin, liver biochemical markers, oxidative stress, sacubitril/valsartan

## Abstract

**Background:**

Cisplatin (CIS) is a highly effective chemotherapeutic agent widely used to treat solid tumors. However, its clinical use is significantly limited by dose-dependent hepatotoxicity, characterized by hepatocellular injury and apoptosis. Despite extensive research efforts, an effective pharmacological strategy to reduce CIS-induced liver dysfunction remains elusive. Sacubitril/valsartan (VS), an angiotensin receptor–neprilysin inhibitor, has shown cytoprotective and anti-apoptotic effects in various models of organ toxicity. However, its ability to protect against CIS-induced liver damage has not been thoroughly studied. This research aimed to assess the hepatoprotective potential of VS in rat models of cisplatin-induced liver toxicity, focusing on oxidative markers including reactive oxygen species (ROS) and malondialdehyde (MDA), as well as the roles of caspase-3 inhibition and modulation of retinoid X receptor-alpha (RXR-α) in its mechanism.

**Methods:**

In this study, adult male Wistar rats were randomly assigned to four groups: control, VS-treated, cisplatin-treated, and CIS + VS co-treated. Hepatotoxicity was induced by administering cisplatin at 8 mg/kg via intraperitoneal injection, repeated over three cycles. Meanwhile, VS was given orally at 60 mg/kg daily for 10 days. Liver biochemical markers, including ROS, MDA, alanine aminotransferase (ALT), aspartate aminotransferase (AST), alkaline phosphatase (ALP), total protein (TP), albumin (ALB), total bilirubin (TBIL), and lactate dehydrogenase (LDH), were measured using ELISA. Liver tissue was examined histologically with hematoxylin and eosin staining, and the expression of caspase-3 and RXR-α was evaluated through immunofluorescence.

**Results:**

Cisplatin administration significantly increased ROS, MDA, ALT, AST, ALP, TBIL, and LDH levels, while decreasing TP and ALB, indicating severe liver dysfunction. Histopathology showed extensive hepatocellular degeneration, necrosis, and inflammation. Co-treatment with VS significantly normalized liver function tests, improved protein levels, and maintained normal liver histology. Additionally, VS markedly reduced caspase-3 immunoreactivity while increasing RXR-α expression compared to CIS alone.

**Conclusion:**

Sacubitril/valsartan appears to protect the liver from cisplatin toxicity, primarily by inhibiting oxidative stress and apoptosis through caspase-3 suppression, and modulating RXR-α signaling. These results provide new insights into the mechanisms involved and suggest that VS may be a promising adjunct therapy to lessen cisplatin-induced hepatotoxicity during chemotherapy.

## 1 Introduction

Cancer continues to be a predominant cause of mortality globally, with chemotherapy serving as a pivotal component in its management (Alhowail, 2025; Alsaud et al., 2023). Among chemotherapeutic agents, cisplatin (CIS) is extensively utilized due to its efficacy against solid tumors, including those in testicular, ovarian, and lung cancers. Nevertheless, its clinical application is considerably limited by severe dose-dependent toxicities, primarily affecting the liver, kidneys, and heart (Aldubayan, 2025b; Alotayk et al., 2023). CIS induces toxic effects across various tissues, manifesting as ototoxicity (75%–100%), nephrotoxicity (72%) (Bunel et al., 2017), hepatotoxicity (36%) (Al-Malki and Sayed, 2014), and cardiotoxicity (6%) (Hu et al., 2018). Hepatotoxicity associated with CIS administration remains a critical clinical issue, as hepatic dysfunction can impair drug metabolism, intensify systemic toxicity, and necessitate dose reduction or cessation of therapy. The mechanisms underlying cisplatin-induced hepatotoxicity are multifaceted, involving oxidative imbalance, inflammatory cascades, mitochondrial dysfunction, and apoptotic cell death within hepatocytes (Hassan et al., 2020; Wu et al., 2025).

Oxidative stress constitutes a fundamental and early event in cisplatin-induced hepatotoxicity, resulting from the excessive generation of reactive oxygen species (ROS) and the impairment of endogenous antioxidant defense mechanisms (Fathy et al., 2022). Cisplatin disrupts mitochondrial respiratory function and promotes electron leakage, leading to excessive ROS production and lipid peroxidation within hepatocyte membranes. This oxidative imbalance compromises cellular integrity, perturbs calcium homeostasis, and activates redox-sensitive signaling pathways, including inflammatory mediators and apoptotic cascades (Raouf et al., 2025). In hepatic tissue, sustained oxidative stress further exacerbates mitochondrial dysfunction and sensitizes hepatocytes to programmed cell death, thereby amplifying liver injury. Collectively, these processes underscore oxidative stress as a pivotal upstream mechanism that links cisplatin exposure to inflammation, apoptosis, and subsequent fibrotic remodeling in the liver (Man et al., 2020; Oliveira et al., 2024).

Apoptosis is a critical factor in the hepatocellular damage caused by CIS. The initiation of pro-apoptotic mediators, particularly caspase-3, marks a significant terminal stage in the apoptotic process, leading to DNA fragmentation and disruption of membrane integrity (Lu and Cederbaum, 2006; Taghizadeh et al., 2021; Wu et al., 2025). Studies have shown that caspase-3 expression is elevated in liver tissues following CIS exposure, which is associated with extensive hepatocellular necrosis and impaired liver function (Bekhit et al., 2023; Lu and Cederbaum, 2006; Wu et al., 2025). Consequently, modulating apoptotic pathways, particularly by regulating caspase-3 activity, could be a viable strategy to reduce cisplatin-induced liver injury.

Beyond its involvement in apoptosis, nuclear receptor signaling plays a crucial role in maintaining hepatic homeostasis and mediating responses to xenobiotic injury. The Retinoid X Receptor-Alpha (RXR-α), a member of the nuclear receptor superfamily, functions as a transcriptional regulator by forming heterodimers with other nuclear receptors, including peroxisome proliferator-activated receptors and retinoic acid receptors. RXR-α is integral to various physiological functions, including lipid metabolism, inflammation, and cell survival (Gyamfi et al., 2008). Research has demonstrated that changes in RXR-α expression or activity are linked to hepatic inflammation and apoptosis. Restoring RXR-α signaling has been shown to confer hepatoprotective benefits in various toxic and inflammatory scenarios (Abdel-Bakky et al., 2021; Xu et al., 2024). Despite these findings, its specific contribution to cisplatin-induced liver injury remains poorly characterized.

The biochemical analysis of serum liver function markers is crucial for understanding the extent of hepatocellular damage and the liver’s metabolic capabilities. Elevated levels of alanine aminotransferase (ALT), aspartate aminotransferase (AST), and alkaline phosphatase (ALP) are indicative of hepatocellular and biliary injury. In contrast, increased total bilirubin (TBIL) and lactate dehydrogenase (LDH) levels suggest compromised hepatic clearance and membrane integrity. On the other hand, decreased levels of total protein (TP) and albumin (ALB) point to a reduced synthetic capacity and overall hepatic dysfunction (Aubrecht et al., 2013; Thakur et al., 2024). These biomarkers collectively provide sensitive measures for evaluating cisplatin-induced hepatotoxicity and the effectiveness of potential hepatoprotective treatments (Bademci et al., 2021).

Sacubitril/valsartan (VS) represents a novel class of angiotensin receptor–neprilysin inhibitors, approved for the treatment of heart failure with reduced ejection fraction. While its cardiovascular benefits are well-established, emerging research highlights VS’s potential cytoprotective, antioxidative, anti-inflammatory, and anti-apoptotic effects across multiple organ systems (Pascual-Figal et al., 2021). Recent evidence suggests that VS confers protective effects by mitigating oxidative stress and restoring redox equilibrium, in addition to its anti-apoptotic properties. Experimental investigations have shown that VS diminishes oxidative damage and enhances antioxidant capacity in models of chemotherapy and ischemia-induced organ injury. This indicates that the modulation of oxidative stress may play a role in its cytoprotective effects (Mahmoud Refaie et al., 2023; Mohany et al., 2022).

Importantly, VS has been shown to alleviate CIS-induced cardiotoxicity by lowering caspase-3 expression and promoting redox balance in cardiac tissues (Mahmoud Refaie et al., 2023). Previous research has demonstrated that VS downregulates caspase-3 expression and restores the Bcl-2/Bax balance in diabetic kidney disease, thereby confirming its anti-apoptotic potential via the caspase-3/Bax/Bcl-2 axis (Mohany et al., 2022). These findings underscore VS’s ability to protect against chemotherapy-induced tissue injury by modulating apoptotic and inflammatory pathways. However, despite promising results in renal and cardiac models, the protective potential of VS against cisplatin-induced hepatic injury remains to be elucidated. Consequently, this study aims to examine the hepatoprotective effects of VS against CIS-induced hepatotoxicity in rats by assessing caspase-3-mediated apoptosis and RXR-α signaling pathways.

In conclusion, CIS-induced multi-organ toxicity poses a substantial challenge in oncological treatment, necessitating the development of effective protective strategies. VS, with its dual mechanisms of action, shows promise as a novel therapeutic intervention. This study aims to provide a comprehensive understanding of the protective effects of VS, offering new hope for patients undergoing chemotherapy based on CIS. This research could pave the way for its clinical application as an adjunct therapy to mitigate CIS-induced toxicity.

## 2 Materials and methods

### 2.1 Materials

Cisplatin at a concentration of 1 mg/mL was procured from EBEWE Pharma GmbH. mbH, Nfg. KG (Unterach am Attersee, Austria). The VS (Entresto®, 100 mg film-coated tablets) was acquired from Novartis Pharma AG, Basel, Switzerland. The mouse monoclonal RXR-α antibody was sourced from Santa Cruz Biotechnology (TX, United States), while the rabbit polyclonal Anti-Caspase-3 antibody was obtained from Abcam Biotechnology (Cambridge, United Kingdom). Cyanine red (Cy3) conjugated goat anti-rabbit and Alexa 488 conjugated goat anti-mouse antibodies were purchased from Invitrogen (CA, United States). DAPI (4′,6-diamidino-2-phenylindole) was sourced from Sigma Aldrich (MO, United States). The fluoromount® mounting solution was obtained from DAKO (Carpinteria, CA, United States).

### 2.2 Animals

The study incorporated 40 male Wistar rats, each weighing between 200 and 380 g and aged 10–12 weeks, sourced from the College of Pharmacy Animal House at Qassim University, Saudi Arabia. The rats were housed in propylene cages, with four rats per cage, and maintained at a controlled temperature of 25 °C ± 2 °C under a 12-h light-dark cycle. They had unlimited access to food and water. Body weight and mortality rates were monitored daily, and behavioral assessments were conducted during the light cycle. The experimental procedures were sanctioned by the Deanship of Graduate Studies and Scientific Research at Qassim University (Approval No. 25–03–16) and adhered to the National Institute of Health guidelines for the Care and Use of Laboratory Animals.

### 2.3 Experimental groups and treatment schedule

The animals were randomly allocated into four groups (n = 10 each): Control, CIS, VS, and CIS + VS. The control group received an equivalent volume of normal saline by oral gavage once daily for 10 days. The CIS group received intraperitoneal CIS on days 1, 4, and 7 at a dosage of 8 mg/kg per injection over 10 days, with treatments administered every 3 days for a total of 3 cycles (Aldubayan, 2025a; Alhowail, 2025). Prior to each cisplatin administration, individual rats were weighed, and the required dose was calculated on a mg/kg basis (8 mg/kg) using the most recent body weight. The injection volume was calculated according to the prepared cisplatin working solution using the formula: Injection volume (mL) = [Body weight (kg) × 8 mg/kg] ÷ [cisplatin concentration (mg/mL)]. Injection volumes were recalculated before each dosing session to ensure accurate and consistent cisplatin delivery throughout the experimental period. The VS group received Entresto (100 mg per tablet), prepared fresh daily before administration. The tablets were finely powdered and suspended in 10 mL of distilled water to achieve a final concentration of 100 mg/10 mL. The dosage for each rat was individually determined based on body weight, with each animal receiving 60 mg/kg/day via oral gavage for 10 days (Burke et al., 2019). The combination group received CIS (8 mg/kg) via intraperitoneal injection on the first day, then in three cycles every 3 days, and VS (60 mg/kg) orally once daily for 10 days.

### 2.4 Mortality rate and body weight

Continuous evaluation of mortality rates was essential for advancing the study. Cage maintenance was conducted twice daily to facilitate the immediate removal of any deceased animals. Daily body weight assessments were systematically conducted to monitor overall health, enabling early detection of subtle changes and prompt identification of potential health issues.

### 2.5 Blood plasma separation and enzyme-linked immunosorbent assays

Blood samples from the seven animals in each group were obtained via retro-orbital puncture and placed into heparinized tubes. Plasma was isolated by centrifuging the samples at 4,000 rpm for 10 min, after which it was transferred to new tubes. The blood plasma was subsequently utilized to assess liver injury by quantifying liver enzymes, including ALT (Cat. no. RK06385), AST (Cat. no. RK03516), ALP (Cat. no. RK03487), TP (Cat. no. MBS3808613), ALB (Cat. no. RK09134), TBIL (Cat. no. MBS730053), and lactate, LDH (Cat. no. A7625), using a commercially available rat ELISA kit (ABclonal, Woburn, MA, United States) in accordance with the manufacturer’s protocols. Absorbance measurements for each well were conducted at 450 nm utilizing a Microplate Reader (BioTek Instruments, Winooski, VT, United States).

### 2.6 Extraction of rat liver tissue

On day 10, seven animals per group were evaluated to ensure parity among the groups. Following the death of the rats, they were removed, placed in a glass chamber, and anesthetized with CO2 (Alsikhan et al., 2023) before euthanasia via decapitation. Subsequently, the rat livers were excised to produce hepatic tissue homogenates,1 g of hepatic tissue was homogenized in an ice-cold PBS (pH 7.4) using a Qsonica homogenizer (30 Hz, Newtown, CT, United States), the homogenates were centrifuged at 1,000 × *g* for 15 min at 4 °C and the clear supernatants were removed and stored at −20 °C to be used later for the estimation of hepatic ROS (Cat no. RK15283; ABclonal) and MDA (Cat no. RK15281; ABclonal) using ELISA assay. Another portion of the liver was collected and fixed in 10% neutral buffered formalin for histopathological and immunofluorescence studies.

### 2.7 Histopathological evaluation and scoring of liver injury

Liver sections were preserved in 10% neutral buffered formalin, followed by dehydration through a graded ethanol series, clearing in xylene, and embedding in paraffin. They were then stained with hematoxylin and eosin (H&E). The assessment of hepatic injury was conducted using the Suzuki scoring system (Toledo-Pereyra et al., 1993), which examines three criteria: sinusoidal congestion, cytoplasmic vacuolization, and hepatocellular necrosis. The scoring system ranges from 0 (no injury) to 4 (severe injury). All slides were evaluated in a blinded fashion, and the average score was calculated for analysis.

#### 2.7.1 Trichrome stain

The tissue samples underwent deparaffinization, followed by incubation in heated Bouin’s buffer. Subsequently, they were washed with running tap water and incubated with hematoxylin for 3 minutes. After washing, the slides were incubated with Biebrich Scarlet-Acid Fuchsin for 6 minutes and gently rinsed with distilled water. The slides were then incubated in Phosphotungstic/Phosphomolybdic Acid buffer for 6 min, followed by an additional 6 min in aniline blue buffer. After a 1-min incubation with 1% acetic acid, the slides were rinsed with distilled water, dehydrated with 100% ethanol for 30 s, cleared using xylene, and mounted with a mounting solution (Mukhopadhyay et al., 2016).

### 2.8 Immunofluorescence analysis of cleaved caspase 3 and RXR-a

The sections were placed in an oven at 60 °C to facilitate paraffin melting, followed by two 10-min xylene treatments. The slides were rehydrated through a series of graded ethanol solutions and then heated in citrate buffer (pH 6.0) in a microwave for 20 min. This was followed by fixation in 100% methanol. After washing with 0.05% Tween in PBS, the slides were blocked with a PBS buffer containing bovine serum albumin and horse serum for 50 min. Subsequently, the slides were washed and incubated with primary antibodies, either rabbit polyclonal cleaved caspase 3 or mouse monoclonal RXR-α, for 2 h at 37 °C, followed by overnight incubation at 4 °C. The sections were treated with secondary antibodies, either Cyanine red (Cy3) conjugated goat anti-rabbit or Alexa 488-conjugated goat anti-mouse, for 45 min. DAPI (4′,6-diamidino-2-phenylindole) was applied for 2 min to stain the nuclei, and the slides were mounted using a fluorescence mounting solution. Images were captured using a fluorescence microscope (Model: Leica DM 5500B, Leica Microsystems, Wetzlar, Germany). A minimum of six images per section, from at least three rats per group, were analyzed using ImageJ software (NIH, United States) (Abdel-Bakky et al., 2020).

### 2.9 Statistical analysis

The data analysis was conducted using GraphPad Prism 10 software (GraphPad Software, La Jolla, CA, United States). A one-way ANOVA was performed, followed by the Tukey–Kramer test to manage multiple comparisons. A p-value of less than 0.05 was considered to indicate statistical significance. Histopathological injury scores, obtained using the Suzuki scoring system (0–4), were treated as ordinal data. The group comparisons were performed using the Kruskal–Wallis test, followed by Dunn’s *post hoc* test.

## 3 Results

### 3.1 Effect of CIS and VS on body weight and mortality

#### 3.1.1 Survival was monitored daily, and deceased animals were promptly removed

**FIGURE 1:**
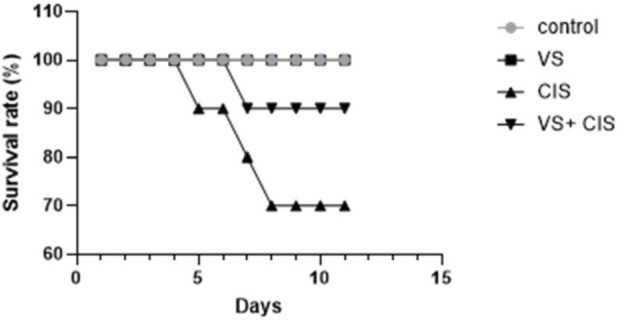
Survival rate of rats following CIS treatment, with or without VS co-administration.

Body weight was recorded daily to assess overall health status and identify any adverse effects associated with the treatment. Cisplatin treatment resulted in a 30% mortality rate by day 10, whereas the co-administration of VS reduced mortality to 10%, compared to 0% in the control group (Figure 1).

**FIGURE 2:**
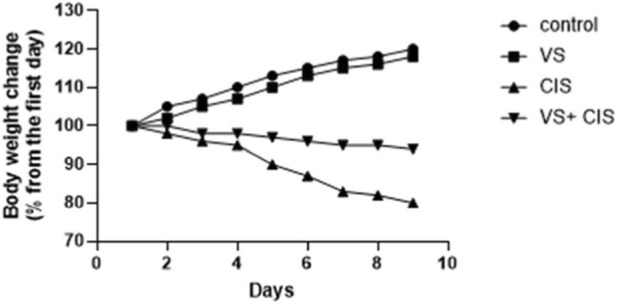
Effect of CIS, VS, and VS + CIS on the body weight.

CIS-treated rats exhibited a reduction in body weight from days 4–9 compared to both the control and CIS + VS groups (Figure 2).

### 3.2 Histopathological staining and scoring of liver injury

**FIGURE 3:**
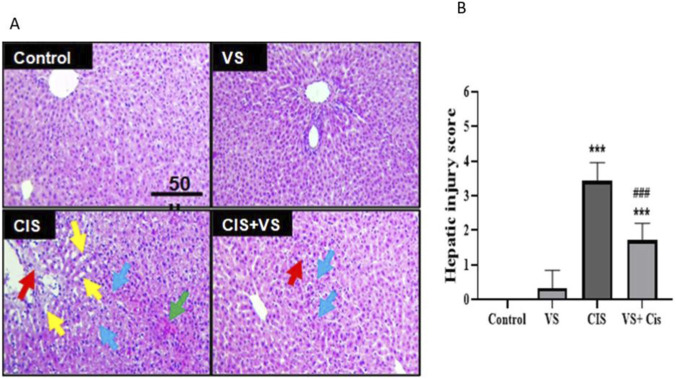
Histopathological assessment of hepatic tissues from rats subjected to CIS and VS treatment was conducted using hematoxylin and eosin staining (H&E, **A)** and evaluated through a hepatic injury score **(B)**. The control and VS-treated rat tissues exhibited normal architectural features. In contrast, CIS-treated rats demonstrated pericentral necrotic areas (indicated by blue arrows), sinusoidal congestion (red arrows), cytoplasmic vacuolization (yellow arrows), and hemorrhage (green arrow). Rats treated with VS showed amelioration of the pathological alterations induced by CIS. Data are presented as mean ± SEM (n = 7). Statistical analysis was performed using the Suzuki scoring system, and the histopathological injury scores were performed using the Kruskal–Wallis test, followed by Dunn’s *post hoc* test. ***p < 0.001 indicates a significant difference compared to control rats, and ###p < 0.001 indicates a significant difference compared to CIS-treated rats. The scale bar represents 50 μm, and the magnification power is ×200.

In the livers of rats from both the control and VS groups, no signs of hepatic injury were detected. In contrast, treatment with CIS led to hepatic damage in both the pericentral and midzonal regions. The pericentral area exhibited more severe effects, including sinusoidal congestion, cytoplasmic vacuolization, disruption of sinusoidal structure, and hepatocellular necrosis. Co-administration of CIS with VS resulted in improved tissue architecture and minimal pathological changes (Figure 3A). The combination of VS with CIS significantly lowered liver injury scores compared to those seen with CIS treatment alone (Figure 3B).

#### 3.2.1 Trichrome stain of rat liver tissues

**FIGURE 4:**
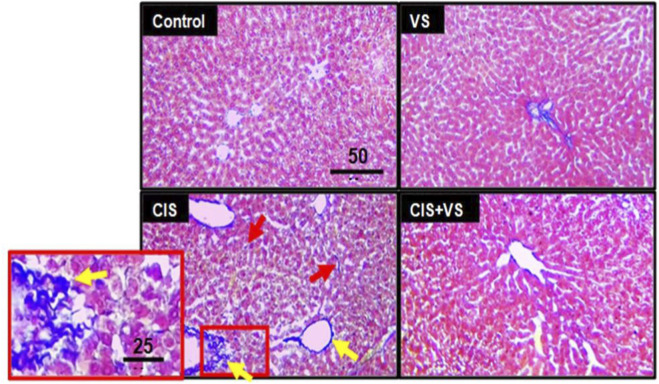
Extracellular matrix in rat liver tissues. Representative histological images illustrating extracellular matrix deposition in liver sections from control, VS, CIS, and VS + CIS-treated rats. Marked accumulation of extracellular matrix is observed in the CIS group, predominantly around the central vein (yellow arrows) and within sinusoidal hepatic stellate cells (red arrows), whereas minimal deposition is detected in control and VS-treated groups. Co-treatment with VS attenuated extracellular matrix accumulation compared with the CIS group. The scale bar measures 50 μm, with a 25 µm scale for the red zoomed box. The images were captured at ×200 magnification.

The Mason trichrome stain of rat liver tissues from both control and VS groups exhibited basal constitutive collagen deposition. In contrast, CIS-treated rats demonstrated significant collagen deposition in the vicinity of the central vein (indicated by yellow arrows) and within the sinusoidal hepatic stellate cells (indicated by red arrows) when compared to normal rat tissues. The VS + CIS group showed a notable reduction in extracellular collagen deposition (Figure 4).

### 3.3 Impact of VS in conjunction with CIS on oxidative stress markers in rat liver tissue

**FIGURE 5:**
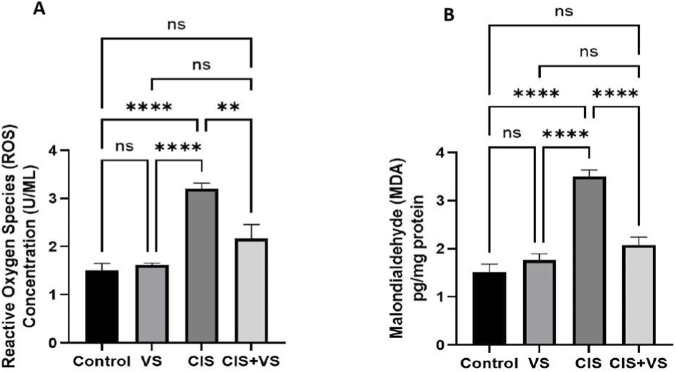
Effect of VS in combination with CIS on ROS and MDA levels. **(A)** Influence of VS on ROS levels in CIS. **(B)** Influence of VS with CIS on MDA levels. The data are reported as mean ± SEM (n = 7). Statistical analysis was conducted using one-way ANOVA, followed by the Tukey–Kramer *post hoc* test. Significance was assessed at **p < 0.01 and ****p < 0.0001 relative to the control and CIS-treated groups.

ROS and MDA levels were significantly higher in the CIS-treated group than in the control and VS + CIS-treated groups. Conversely, VS co-administered with CIS significantly decreased ROS and MDA levels compared with the CIS-treated groups and did not significantly alter ROS or MDA levels compared with the VS-treated alone and the control (Figures 5A,B).

### 3.4 Immunofluorescence analysis of cleaved caspase 3 and RXR-a

#### 3.4.1 Effect of CIS in the presence or absence of VS on cleaved caspase 3 (c-caspase 3) protein expression in rat liver tissues using the immunofluorescence technique

**FIGURE 6:**
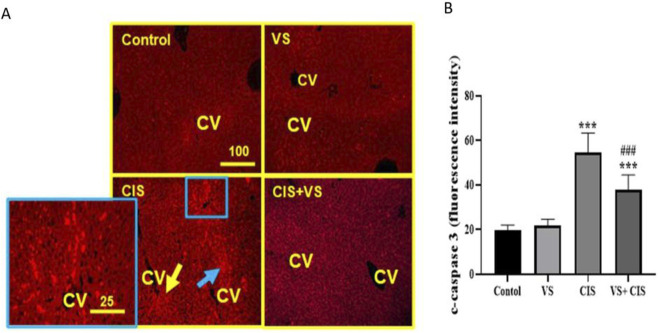
The effect of CIS, in the presence or absence of VS, on cleaved caspase 3 (c-caspase 3) protein expression in rat liver tissues was assessed using the immunofluorescence technique **(A)**, and the fluorescence intensity was quantified and presented in a graph **(B)**. Basal c-caspase 3 protein expression in control and VS-treated groups was observed in the sinusoidal pericentral space. Rats treated with CIS exhibited strong expression of c-caspase 3 protein in the pericentral hepatocytes (indicated by yellow arrows) and in HSCs (indicated by blue arrows). A significant decrease in c-caspase 3 protein was found in the midzonal areas, but not in hepatocytes, compared to CIS-treated rats. Data are presented as mean ± SEM (n = 7). Statistical analysis was conducted using one-way ANOVA followed by the Tukey-Kramer multiple comparisons test, where ***p < 0.001 indicates significant differences compared to control rats, and ###p < 0.001 indicates significant differences compared to CIS-treated rats. The scale bar represents 100 µm with a magnification of ×100. CV denotes the central vein.

Immunofluorescence analysis of liver tissues indicated minimal expression of c-caspase-3 proteins in both control and VS-treated rats. In contrast, liver tissues from CIS-treated rats showed a significant increase in c-caspase-3 expression in pericentral hepatocytes and hepatic stellate cells (HSCs). The VS + CIS group showed reduced c-caspase-3 expression compared with the CIS-treated group (Figure 6A). Fluorescence intensity was measured and plotted for caspase-3 (Figure 6B) using ImageJ/NIH software.

#### 3.4.2 Effect of CIS in the presence or absence of VS on retinoid X receptor-alpha (RXR-α) expression in rat liver tissues using the immunofluorescence technique

**FIGURE 7:**
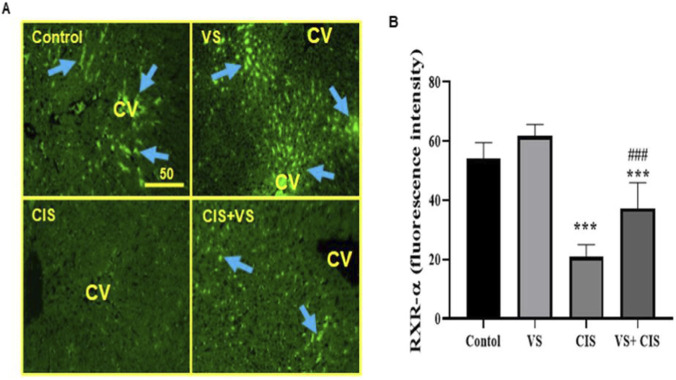
The effect of CIS with or without VS on the expression of RXR-α protein is depicted through green fluorescence **(A)**, alongside a graph illustrating fluorescence intensity **(B)** in rat liver tissues. Data are presented as Mean ± SEM (n = 7), with ***P < 0.001 indicating a significant difference compared to normal rat tissues, and ###P < 0.001 denoting a significant difference when compared to CIS-treated rats, as determined by one-way ANOVA and Tukey Kramer for multiple comparisons among all group means. Scale bar = 50 μm. CV denotes the central vein. Magnification power is ×200.

In control and VS liver tissues, RXR-α is constitutively expressed mainly in the sinusoidal space adjacent to the central vein within HSCs, as indicated by blue arrows. Rats treated with CIS showed a marked reduction in RXR-α expression compared with the control group. Liver tissues from the VS + CIS group exhibited a partial recovery of RXR-α protein in the sinusoidal space around the central vein in HSCs (blue arrows, Figure 7A). The fluorescence intensity data (Figure 7B) further substantiate the suppressive impact of CIS on RXR-α protein expression.

### 3.5 Effect of CIS in the presence or absence of VS on liver function parameters, including AST, ALT, ALP, TIBL, LDH, TP, and ALB levels

**FIGURE 8:**
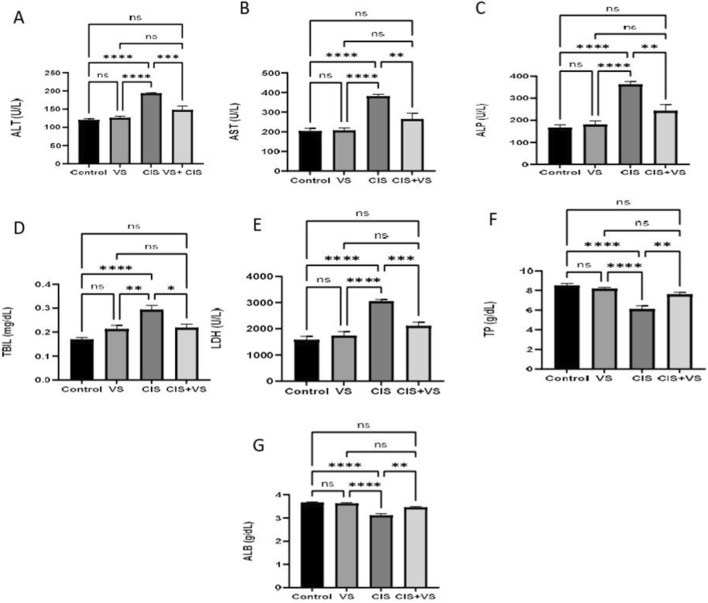
The effect of CIS in combination with VS on hepatic biomarkers was investigated. **(A)** The influence of CIS, both with and without VS, on ALT activity was assessed. **(B)** AST activity was evaluated. **(C)** ALP activity was measured. **(D)** TBIL levels were determined. **(E)** LDH activity was analyzed. **(F)** TP concentrations were quantified. **(G)** ALB concentrations were examined. The data are presented as mean ± SEM (n = 7). Statistical analysis was conducted using one-way ANOVA, followed by the Tukey–Kramer *post hoc* test. Significance was determined at levels of *p < 0.05, **p < 0.01, ***p < 0.001, and ****p < 0.0001 compared with the control and CIS-treated groups.

The administration of CIS resulted in a significant increase in plasma levels of AST, ALT, ALP, TIBL, and LDH compared with the control, VS-treated, and VS + CIS co-treated groups (Figures 8A–E). In contrast, CIS significantly decreased TP and ALB levels relative to the control, VS-treated, and VS + CIS co-treated groups (Figures 8F,G).

## 4 Discussion

In this study, we evaluated the efficacy of VS treatment in alleviating CIS-induced hepatotoxicity in rats through comprehensive *in vivo* experiments that included assessment of hematologic and liver function biomarkers. Furthermore, we investigated the mechanisms underlying VS-mediated mitigation of CIS-induced hepatotoxicity, with a particular focus on modulation of anti-apoptotic pathways and regulation of RXR-α protein expression in rat liver tissues. Rats subjected to CIS treatment exhibited a 30% survival mortality rate by day 10. In contrast, the CIS co-administration with VS reduced mortality to 10%, compared to 0% in the VS-treated alone and control groups. There was a reduction in final body weight compared to the control group. This observation is consistent with previous research, which indicates that although rats resumed weight gain after cessation of CIS treatment, their overall growth rate remained lower than that of control rats (Mokhtar et al., 2021). Cisplatin administration is commonly associated with weight loss, primarily due to its emetogenic effects, which lead to reduced appetite, gastrointestinal toxicity, and diarrhea (He et al., 2023). The administration of VS increased body weight, potentially attributable to its anti-apoptotic properties.

The present study demonstrated that the administration of CIS in rats resulted in significant hepatic injury, as evidenced by both histopathological alterations and elevated biochemical markers indicative of liver dysfunction. Notably, VS treatment substantially ameliorated these deleterious effects, suggesting a hepatoprotective effect mediated through anti-oxidative and anti-apoptotic mechanisms. Histological analysis with H&E staining revealed that CIS induced significant hepatic damage, predominantly affecting the pericentral and midzonal regions. This damage was characterized by sinusoidal congestion, vacuolar degeneration, disruption of hepatic cords, and hepatocellular necrosis. The current findings are consistent with previous studies showing that cisplatin preferentially damages centrilobular hepatocytes, which contain high levels of cytochrome P450, making them more vulnerable to oxidative stress (Abdel-Daim et al., 2019; Palipoch et al., 2014).

Mason trichrome staining revealed further evidence of increased collagen deposition around the central vein and sinusoidal spaces, indicative of early fibrotic remodeling driven by HSC activation (Bataller and Brenner, 2005; Hassan et al., 2020). In contrast, the co-administration of VS with CIS significantly enhanced hepatic architecture, reduced congestion, and minimized necrosis. Additionally, collagen accumulation was substantially diminished in the VS + CIS group, indicating a suppression of extracellular matrix deposition and inhibition of HSC activation. These findings suggest that VS mitigates CIS-induced liver fibrosis, potentially through the modulation of the angiotensin II and TGF-β/Smad pathways, both of which are recognized mediators of stellate cell-driven fibrogenesis (An et al., 2024; Suzuki et al., 2023; Zhang et al., 2021). Angiotensin II (Ang II) plays a crucial role in the development of cisplatin-induced hepatotoxicity and fibrogenesis. While traditionally recognized for its vasoactive properties, Ang II also acts as a significant pro-oxidant, pro-inflammatory, and pro-fibrotic agent in liver tissue (Bataller et al., 2000). Cisplatin exposure has been shown to activate the hepatic renin–angiotensin system, thereby increasing Ang II signaling. This activation results in the generation of reactive oxygen species via NADPH oxidase, activation of NF-κB, and the release of pro-inflammatory cytokines, thereby worsening liver cell damage (Bataller et al., 2000; Macêdo et al., 2014; Ozkok and Edelstein, 2014). Additionally, Ang II directly triggers hepatocyte apoptosis through mitochondrial dysfunction and caspase-3 activation. It also promotes the activation of hepatic stellate cells and the deposition of extracellular matrix via the TGF-β/Smad signaling pathway, thereby accelerating fibrotic changes (Bataller and Brenner, 2005; Dewidar et al., 2019). Sacubitril/valsartan mitigates these pathological mechanisms through complementary pharmacological actions. Valsartan inhibits the binding of Ang II to the AT_1_ receptor, thereby reducing Ang II-mediated oxidative stress, inflammatory signaling, apoptosis, and stellate cell activation. Simultaneously, sacubitril inhibits neprilysin, leading to increased circulating and local natriuretic peptide levels, which exert anti-inflammatory, anti-apoptotic, and anti-fibrotic effects in hepatic tissue (Suzuki et al., 2023). The combined suppression of Ang II–driven injury pathways and enhancement of protective natriuretic peptide signaling provides a mechanistic basis for the observed reduction in caspase-3 activation, attenuation of collagen deposition, and preservation of hepatic architecture in the VS + CIS group.

Oxidative stress represents a pivotal early mechanism in cisplatin-induced hepatotoxicity and serves as a critical trigger for subsequent apoptotic and fibrotic responses (Aboraya et al., 2022). In the present study, CIS administration markedly increased hepatic ROS generation and MDA levels, indicating excessive oxidative burden and enhanced lipid peroxidation. These findings are consistent with previous reports demonstrating that cisplatin disrupts hepatic redox homeostasis by promoting ROS overproduction and membrane lipid damage, thereby exacerbating hepatocellular injury (Abdel-Daim et al., 2019; Aboraya et al., 2022). Importantly, co-treatment with the VS significantly attenuated the CIS-induced elevation of ROS and MDA, indicating partial restoration of hepatic redox balance. This antioxidative effect of VS likely contributes to the observed preservation of hepatic architecture, suppression of caspase-3–mediated apoptosis, and attenuation of extracellular matrix deposition. Collectively, these results suggest that modulation of oxidative stress constitutes a potential mechanism underlying the hepatoprotective effects of VS against cisplatin-induced liver injury.

Beyond the oxidative stress effect, immunofluorescence analysis demonstrated pronounced expression of c-caspase-3 in pericentral hepatocytes and HSCs of rats administered CIS, indicating activation of apoptotic mechanisms. This observation is consistent with previous research, which has shown that CIS induces apoptosis in hepatocytes by disrupting the mitochondrial Bax/Bcl-2 equilibrium and activating caspase-3 (Hassan et al., 2020). In contrast, the combined treatment with VS and CIS significantly reduced c-caspase-3 expression, suggesting that VS inhibits caspase-dependent apoptosis. This anti-apoptotic effect of VS has been previously documented in cardiac and renal models of cisplatin toxicity, in which VS downregulated caspase-3 by suppressing NF-κB (Mahmoud Refaie et al., 2023; Mohany et al., 2022; Yadav, 2015). Therefore, the hepatoprotective effect observed in this study likely involves similar molecular signaling mechanisms.

In addition to its anti-apoptotic effects, VS may also help preserve RXR-α expression in hepatocytes and hepatic stellate cells. RXR-α is a critical nuclear receptor that forms heterodimers with PPARs, LXRs, and FXRs to regulate lipid metabolism, detoxification, and hepatocyte survival (Xu et al., 2024). Previous study reported that depletion of RAR-α and RXR-α was accompanied by enhanced caspase-3–dependent apoptosis in *N-acetyl-p-aminophenol*-induced liver injury, demonstrating a direct association between retinoid receptor loss and apoptotic signaling (Abdel-Bakky et al., 2021). In line with these findings, the partial restoration of RXR-α expression observed in the VS + CIS group suggests that VS may help maintain retinoid receptor integrity, thereby limiting caspase-3–mediated apoptosis and contributing to overall hepatocellular protection.

The biochemical modifications identified in this study provide additional support for the histopathological and molecular observations. The administration of CIS resulted in pronounced elevations in AST, ALT, ALP, TBIL, and LDH, along with significant reductions in total TP and ALB. These biochemical parameters serve as indicators of hepatocellular injury, cholestasis, and impaired hepatic synthetic function (Abd Rashid et al., 2021; Palipoch and Punsawad, 2013). It is crucial to recognize that serum albumin and total protein have relatively long half-lives, approximately 19–21 days. As a result, the reductions observed during this short-term experimental period may not exclusively reflect a compromised hepatic synthetic capacity (Levitt and Levitt, 2016). Additionally, cisplatin-induced nephrotoxicity has been shown to increase urinary protein loss, which may contribute to the observed decrease in circulating protein levels (Alharbi and Aldubayan, 2025; Levitt and Levitt, 2016; Ozkok and Edelstein, 2014). Therefore, the reductions in total protein and albumin in this study should be interpreted as systemic effects of cisplatin-induced toxicity rather than isolated signs of hepatic synthetic dysfunction. These modifications are consistent with prior research indicating that it reduces TP and ALB levels following cisplatin administration, indicative of membrane leakage and hepatocyte necrosis (Palipoch and Punsawad, 2013; Yadav, 2015). Notably, the co-administration of VS successfully restored these biochemical parameters to normal levels, indicating a potential protective role in maintaining hepatocellular integrity and metabolic function. This positive outcome is consistent with the observed preservation of hepatic architecture and the decreased caspase-3 expression in the VS + CIS group. Additionally, the simultaneous restoration of RXR-α expression in hepatocytes suggests that VS may enhance nuclear receptor–mediated regulatory pathways, which are crucial for liver function and cell survival, thereby augmenting its hepatoprotective potential.

A key strength of this study is its novel demonstration of the hepatoprotective effects of VS in mitigating CIS-induced hepatotoxicity. The study offers novel mechanistic insights into the anti-apoptotic effects of VS, as evidenced by histopathological, biochemical, and immunofluorescence analyses that show reduced caspase-3 activation and partial restoration of RXR-α expression. This comprehensive evaluation reinforces the causal relationship between VS treatment and protection against apoptosis-mediated hepatic injury. However, several limitations of this study warrant acknowledgment. The investigation primarily concentrated on apoptotic markers, omitting the assessment of oxidative stress and inflammatory mediators, which are also critical contributors to CIS-induced liver injury. Furthermore, the absence of a valsartan-only treatment group precludes distinguishing the specific effects of valsartan from the combined actions of VS. Future research should incorporate these parameters, alongside molecular signaling analyses, to comprehensively elucidate the mechanistic basis and clinical potential of VS for hepatic protection. In addition, although the current findings indicate a strong association between VS treatment and reduced caspase-3 activation, the causal role of caspase-3 inhibition was not confirmed using selective caspase-3 inhibitors or activators. Furthermore, other significant molecular contributors to cisplatin-induced liver injury, such as NF-κB-mediated inflammatory signaling and the TGF-β/Smad pathway, were not directly assessed. These pathways are well recognized for their roles in regulating cytokine production, hepatic stellate cell activation, and extracellular matrix deposition during cisplatin-induced hepatotoxicity (Gazwi et al., 2024). Although the 10-day experimental duration is adequate to induce acute cisplatin-related liver injury (Eisa et al., 2021; Gholampour et al., 2022) and to demonstrate the hepatoprotective effects of VS, as short-term VS administration has been shown to attenuate apoptosis, inflammation, and fibrotic responses in experimental models of drug combination in an organ-toxicity model (Elemam et al., 2025; Mahmoud Refaie et al., 2023), longer-term studies are necessary to evaluate chronic hepatic outcomes and the sustained hepatoprotective effects of VS, thus warranting further investigation in future studies.

In conclusion, the administration of CIS led to notable structural and biochemical changes in the liver, characterized by significant hepatocellular damage, increased liver enzyme activities, enhanced caspase-3-mediated apoptosis, and decreased RXR-α expression. Co-treatment with VS significantly alleviated these detrimental effects, improving liver architecture, normalizing biochemical parameters, and partially restoring RXR-α expression. Collectively, these findings indicate that VS exerts hepatoprotective effects primarily through its anti-apoptotic action and modulation of RXR-α–associated survival signaling, suggesting its potential as an adjunctive agent against CIS-induced hepatotoxicity.

## Data Availability

The original contributions presented in the study are included in the article/supplementary material, further inquiries can be directed to the corresponding author.
